# Diversity of *Cardinium* Endosymbiont Genomes from Plant-Parasitic Nematodes

**DOI:** 10.3390/ijms27021038

**Published:** 2026-01-20

**Authors:** Sergey V. Tarlachkov, Alexander Y. Ryss, Yury Y. Ilinsky, Dmitry A. Rodionov, Lydmila I. Evtushenko, Sergei A. Subbotin

**Affiliations:** 1 All-Russian Collection of Microorganisms (VKM), G.K. Skryabin Institute of Biochemistry and Physiology of Microorganisms, Pushchino Scientific Center for Biological Research of the Russian Academy of Sciences, Pushchino 142290, Russia; sergey@tarlachkov.ru (S.V.T.); lie99@mail.ru (L.I.E.); 2 Laboratory for Parasitic Worms and Protists, Zoological Institute of the Russian Academy of Sciences, Universitetskaya Naberezhnaya 1, St. Petersburg 199034, Russia; alexander.yu.ryss@gmail.com; 3 Laboratory of Molecular Genetics of Insect, Institute of Cytology and Genetics of Siberian Branch of Russian Academy of Sciences, Novosibirsk 630090, Russia; paulee@bionet.nsc.ru; 4 Sanford Burnham Prebys Medical Discovery Institute, La Jolla, CA 92037, USA; rodionov@sbpdiscovery.org; 5 California Department of Food and Agriculture, Plant Pest Diagnostic Center, Sacramento, CA 95832, USA; 6 Department of Entomology and Nematology, University of California One Shields Avenue, Davis, CA 95616, USA

**Keywords:** bacteria, biotin, horizontal gene transfer, metabolic pathways, phylogeny

## Abstract

*Cardinium* endosymbionts are obligate intracellular bacteria found in a wide range of invertebrate hosts. In this study, we generated ten new *Cardinium* genomes from plant-parasitic nematodes of the genera *Amplimerlinius*, *Bursaphelenchus*, *Cactodera*, *Ditylenchus*, *Globodera*, *Meloidoderita*, and *Rotylenchus*, revealing their broad ecological and phylogenetic distribution. Using an expanded set of genes, we clarified the relationship between previously defined *Cardinium* groups B and F from nematodes, showing that they are closely related and likely share a single evolutionary origin within nematode-associated *Cardinium*. Among the newly assembled *Cardinium* genomes obtained in this study, two genomes originating from strains associated with wood-inhabiting *Bursaphelenchus* species exhibited remarkable genome reduction, with estimated sizes of approximately 695 kb. Functional annotation of *Cardinium* genomes indicated an absence of or a reduction in several central metabolic pathways, including the biotin biosynthetic pathway. A complete biotin pathway was found only in *D. weischeri*, and this pathway is only partially encoded in *Cactodera* sp. The *polA* gene, which encodes DNA polymerase I, showed partial loss in several *Cardinium* strains. Phylogenetic and comparative genomic analyses provided strong evidence that several carbohydrate, glycerophospholipid, and biotin metabolism genes in these endosymbionts have been acquired through horizontal gene transfer. Future research that integrates high-quality genome assemblies with functional analyses of host–symbiont interactions will be essential to elucidate how metabolic dependency, genome reduction, and horizontal gene transfer collectively shape the evolution and ecological diversification of *Cardinium* across nematode hosts.

## 1. Introduction

Bacteria belonging to the genus *Candidatus* Cardinium (Bacteroidota) and related organisms composing the *Cardinium* clade are intracellular endosymbionts that are widely spread among a diverse range of invertebrate hosts. These include arachnids, insects, copepods, ostracods, and mussels, as well as certain plant-parasitic nematodes [1,2,3,4,5,6,7,8]. *Cardinium* is best known for its ability to manipulate host reproduction through a variety of mechanisms: cytoplasmic incompatibility, parthenogenesis induction, and feminization [8]. Endosymbionts can strongly influence an insect host’s nutrition, immune function, development, and reproductive processes [9]. It has been hypothesized that this bacterium might stimulate pheromone production (aggregation/sex) via the terpenoid pathway [10]. *Cardinium* shows promise for applications in the control of arthropod pest species and arthropod-vectored disease transmission; however, much remains unknown about its physiology and interactions with host organisms. Although *Cardinium*-associated phenotypes have been investigated in various arthropod hosts, very little is known about the phenotypic effects of *Cardinium* infection in nematodes [8].

Phylogenetic analyses based on 16S rRNA gene sequences have delineated *Cardinium* into seven groups [4,7]. This grouping is further supported by analyses of additional loci, including *gyrB*, *sufB*, *groEL*, *fusA*, and *infB* [7,11]. Group A is currently the most well characterized and broadly distributed, encompassing strains that infect a wide array of arthropod hosts, including both mandibulate taxa (e.g., insects, crustaceans) and chelicerates (e.g., mites, spiders) [4]. Group B is composed of *Cardinium* strains associated with plant-parasitic nematodes, such as *Heterodera* species, *Globodera rostochiensis*, *Cactodera rosae*, *Punctodera chalcoensis*, and *Rotylenchus zhongshanensis* [4,7,12,13]. Group C appears to be host-restricted, consisting exclusively of *Cardinium* strains isolated from *Culicoides* biting midges [14]. Subsequently, representatives of group D were reported in a single marine copepod species, *Nitocra spinipes*, and presumed to occur in other copepods [15]. Group E contains a *Cardinium* strain from a single oribatid mite species, *Achipteria coleoptrata* [16], whereas group F is composed of that from a plant-parasitic root lesion nematode, *P. penetrans*, only [17]. Group G includes *Cardinium* strains from non-marine ostracod species and a freshwater mussel, *Unio crassus* [6,7,18]. Recently, intracellular *Cardinium*-like symbionts were detected in the wood-inhabiting nematode *Bursaphelenchus mucronatus* through transmission electron microscopy [19]. These endosymbionts were observed within the nematode’s tissues, suggesting a potential intimate association between the microorganism and its host. However, the molecular characterization and phylogenetic identification of this *Cardinium*-like bacterium have not yet been performed, leaving its taxonomic position and functional role within *B. mucronatus* unresolved.

To date, 27 *Cardinium* genomes are publicly available, representing a growing but still limited genomic dataset for this diverse group of endosymbionts. Of these, seven genomes have been completed, and the remaining genomes are draft assemblies of varying quality and remain incomplete [8]. Two genomes belong to *Cardinium* group B, one to group F, and the rest to group A. The *Cardinium* genomes are reduced in size (0.9–1.5 Mbp), with a 33.5–39.0% GC content and a gene count of ~1000 [8,20,21]. For comparison, the closest known relative of *Cardinium*, the species *Candidatus* Amoebophilus asiaticus, which is also an obligate symbiont, has a 1.9 Mbp genome, ~1500 genes, and a 35% GC content [22].

The primary aim of this study was to expand the genomic information on *Cardinium* from plant-parasitic nematodes. To achieve this, we screened both newly generated and publicly available raw genomic sequencing data, successfully recovering ten previously unreported *Cardinium* genomes. We conducted phylogenetic analyses to determine their evolutionary relationships, assembled genomes of ten *Cardinium* strains, and performed annotation to characterize their genomic features. Notably, this study reports the first detection of *Cardinium* in two additional nematode hosts, namely, a stem nematode of the genus *Ditylenchus* and a sedentary nematode of the genus *Meloidoderita*, and also molecularly confirms the presence of *Cardinium* in wood-inhabiting nematodes of the genus *Bursaphelenchus.* These findings broaden the known host range of *Cardinium* and contribute to a more complete understanding of the diversity and evolutionary history of this bacterium within nematode symbioses.

## 2. Results

### 2.1. New Findings of Cardinium in Plant-Parasitic Nematodes and Their Phylogenetic Relationships

Screening of both original and publicly available Sequence Read Archive (SRA) datasets for the presence of *Cardinium* 16S rRNA and *gyrB* gene fragments revealed the occurrence of this endosymbiotic bacterium in genomic assemblies of several nematode taxa. Specifically, *Cardinium* sequences were detected in datasets corresponding to *Amplimerlinius* sp., *Bursaphelenchus fraudulentus*, *B. mucronatus*, *Ditylenchus weischeri*, *Globodera ellingtonae*, and *Rotylenchus zhongshanensis* (Table A1). In addition, the presence of *Cardinium* was confirmed in the SRA datasets of *Cactodera rosae*, *Cactodera* sp., *G. rostochiensis*, *Heterodera goettingiana*, *H. latipons*, and *H. sturhani*, as identified in our previous study [6].

**Figure 1:**
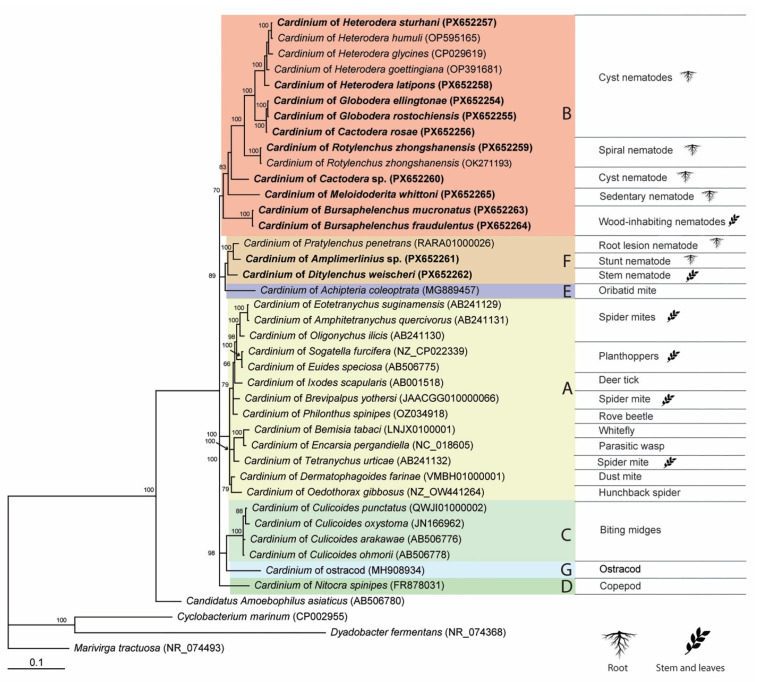
Phylogenetic relationships among representatives of the *Cardinium* clade. Bayesian 50% majority rule consensus tree as inferred from 16S rRNA gene sequence alignment (ntax = 41; nchar = 1468 bp) under the GTR + I + G model. Posterior probabilities of more than 50% are shown at branching points. New sequences are given in bold. Capital letters and color areas indicate the *Cardinium* group, and symbols of root and stem with leaves indicate part of the plant from which the host organism is feeding.

The phylogenetic relationships of *Cardinium* strains based on the 16S rRNA gene sequences obtained from plant-parasitic nematodes and other arthropod hosts are shown in Figure 1. Overall, the relationships among the major *Cardinium* groups were not well resolved based on the 16S rRNA gene, due to the conserved nature of this marker and limited phylogenetic signal. Intraspecific sequence variation for group B was up to 6.5%, group F—2.7%, and group A—3.1%.

**Figure A1:**
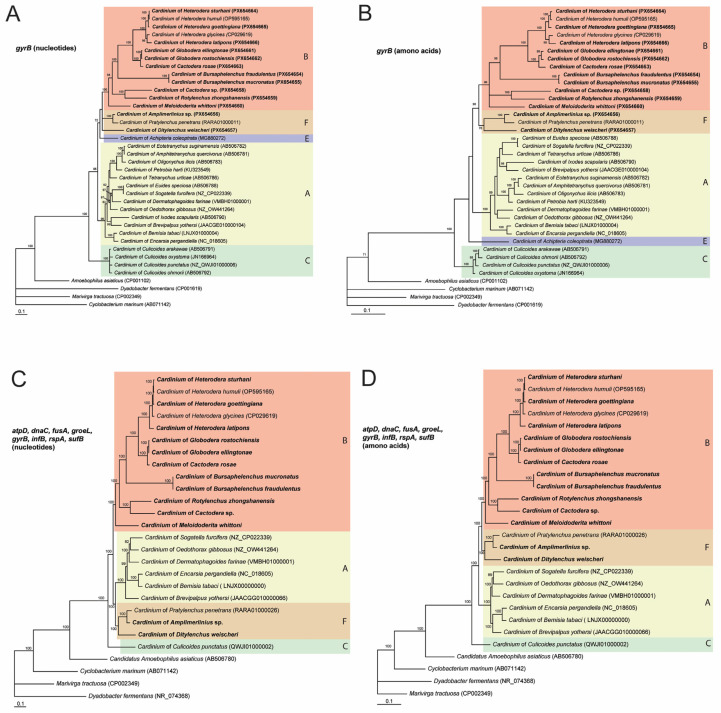
Phylogenetic relationships among representatives of the *Cardinium* clade. Bayesian 50% majority rule consensus trees as inferred from *gyrB* nucleotide (ntax = 39; nchar = 1412) (**A**) and amino acid (ntax = 39; nchar = 470) (**B**). Bayesian 50% majority rule consensus trees as inferred from the concatenation of *atpD*, *dnaB*, *fusA*, *groEL*, *gyrB*, *infB*, *rspA*, and *sufB* genes: nucleotide (ntax = 27; nchar = 12,638) (**C**) and amino acid (ntax = 27; nchar = 4122) (**D**) sequence alignments. Posterior probabilities of more than 70% are shown at branching points. New sequences are given in bold. See Table S2 for GenBank accession numbers. Capital letters and color areas indicate the *Cardinium* group.

**Figure A2:**
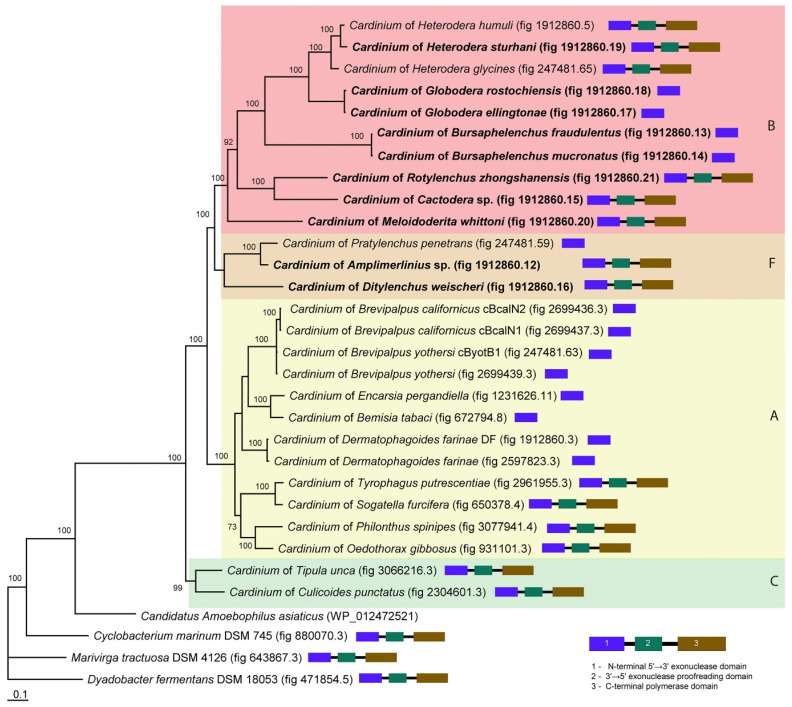
Phylogeny of the *polA* gene with domain mapping as inferred from Bayesian analysis of amino acid sequence alignment (ntax = 33; nchar = 997). Posterior probabilities of more than 70% are shown at branching points. New sequences are given in bold. Capital letters and color areas indicate the *Cardinium* group.

**Figure A3:**
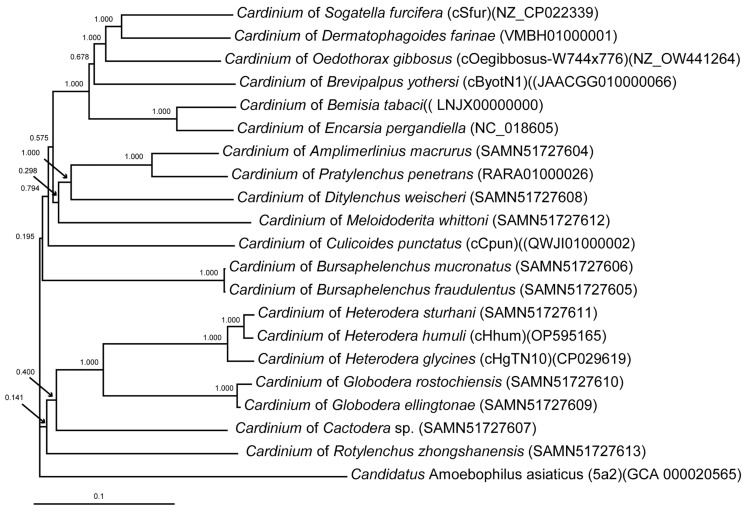
Phylogenomic relationships among representatives of the *Cardinium* clade. The tree is drawn to scale, with branch lengths measured in the estimated evolutionary distance.

Phylogenetic trees inferred from *gyrB* gene fragment sequences and from a concatenated dataset comprising eight protein-coding genes (*atpD*, *dnaB*, *fusA*, *groEL*, *gyrB*, *infB*, *rpsA*, and *sufB*) (Table A2) are presented in Figure A1. These multilocus phylogenetic analyses provided improved phylogenetic resolution within the *Cardinium* clade. Notably, *Cardinium* strains associated with nematodes formed a well-supported monophyletic lineage (groups B and F) in all analyses, except for the tree reconstructed from the nucleotide alignment of the eight-gene concatenation, where the nematode-associated sequences appeared paraphyletic. In single-gene phylogenetic analyses, nucleotide sequences of *dnaB*, *groEL*, *gyrB*, and *rpsA* recovered nematode-associated *Cardinium* as a monophyletic lineage, whereas analyses based on *atpD*, *fusA*, *infB*, and *sufB* indicated paraphyletic relationships. Maximum likelihood comparisons of alternative tree topologies using the Kishino–Hasegawa (KH), Shimodaira–Hasegawa (SH), and Shimodaira approximately unbiased (AU) tests did not reject the hypothesis (*p* < 0.05) of nematode-associated *Cardinium* monophyly for the *atpD*, *fusA*, and *sufB* datasets. Only the *infB* alignment significantly rejected this hypothesis under both SH (*p* = 0.034) and AU (*p* = 0.017) tests. Phylogenetic analysis based on amino acid sequences of the *polA* gene also demonstrated the monophyly of *Cardinium* strains from plant-parasitic nematodes (Figure A2). The sequence of *Cardinium* from biting midges occupied a basal position on the trees in all the analyses. The phylogenomic tree inferred from 20 bacterial genomes of the *Cardinium* clade using JolyTree is given in Figure A3.

**Figure A4:**
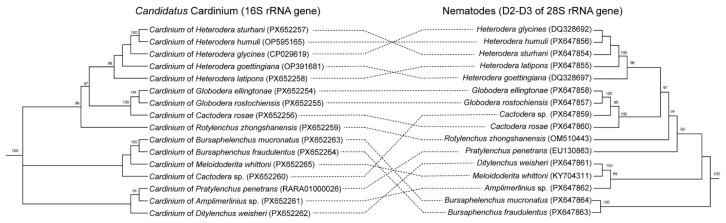
Cophylogenetic relationships between the endosymbionts *Candidatus* Cardinium (16S rRNA gene tree; ntax = 16; nchar = 1468) and their nematode hosts (partial 28S rRNA gene tree; ntax = 16; nchar = 819). Bayesian 50% majority consensus trees with posterior probability values of more than 70% for appropriate clades. Dotted lines show the association between bacteria and their hosts.

The *Cardinium* 16S rRNA gene sequences and the D2–D3 expansion fragments of the nematode 28S rRNA gene were used to perform a cophylogenetic analysis aimed at exploring potential patterns of host–symbiont association. Although the 16S rRNA gene provided limited phylogenetic resolution among *Cardinium* strains, the Procrustean Approach to Cophylogeny (PACo) analysis revealed a significant global cophylogenetic signal, indicating that host and symbiont phylogenies are more congruent than expected by chance. The sum of squared residuals (SS) was 0.78, and the permutation test with 1000 replicates produced a *p*-value of 0, supporting the hypothesis of cophylogeny. However, there were several clear topological incongruences between some bacterial and host lineages (Figure A4). For instance, *Cardinium* sequences associated with wood-inhabiting nematodes of the genus *Bursaphelenchus* clustered together with bacterial strains from sedentary plant-parasitic nematodes, despite the fact that these hosts belong to different nematode superfamilies or orders and are only distantly related. Similarly, two morphologically and genetically similar species of *Cactodera* harbored phylogenetically distinct and unrelated *Cardinium* strains (Figure A4).

### 2.2. The Whole and Draft Genomes of Cardinium and Their Annotation

**Figure 2:**
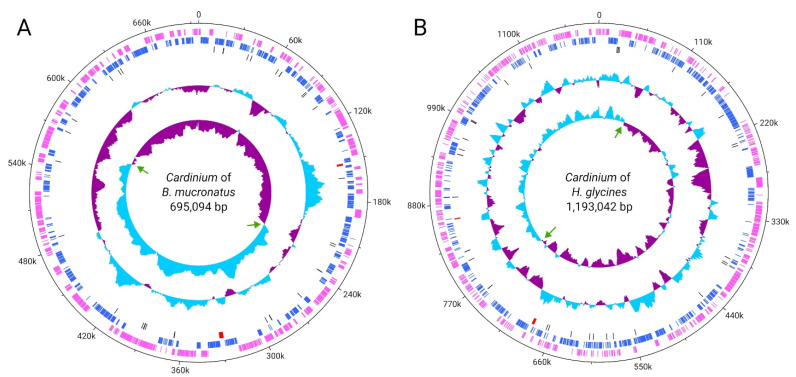
Chromosome maps of *Cardinium* of *Bursaphelenchus mucronatus* (**A**) and *Cardinium* of *Heterodera glycines* (**B**). Designations from the outer circle to the inner one: circles 1 and 2—predicted genes in forward and reverse orientation, respectively, 3—rRNAs, tRNAs, and tmRNAs, 4—GC-plot, showing deviations from the mean value, and 5—GC-skew. Coordinates are specified in bp.

In this study, we successfully assembled ten *Cardinium* genomes, including two complete (single circular contig without gaps) and eight draft assemblies (Table S1). The summary statistics for these genomes are presented in Table 1. The assemblies ranged from 1 to 144 scaffolds, with total sizes varying between 695,026 and 1,345,476 base pairs. The *Cardinium* endosymbionts of *Bursaphylenchus fraudulentus* and *B. mucronatus* have chromosomes of 695,026 bp and 695,094 bp (Figure 2) in size, respectively, which were assembled in a single circular contig (Table 1 and Table S1). Sequencing depth (genome coverage) for these ten genomes spanned from 19× to 1313×. The GC content of the ten genomes ranged from 35.23% to 41.07%. A total of 600 to 1025 protein-coding genes were predicted per genome, reflecting notable variability in gene content across different strains. There was no evidence of plasmids in the genomes. CheckM analysis showed low levels of contamination (less than 5%) and moderate levels of completeness (approximately 70%). It is worth noting that other *Cardinium* genomes represented in Genbank have a similar level of completeness. For example, the complete genome of *Cardinium hertigii* cHgTN10 has a completeness metric of 70% with a contamination level of 1%.

**Figure A5:**
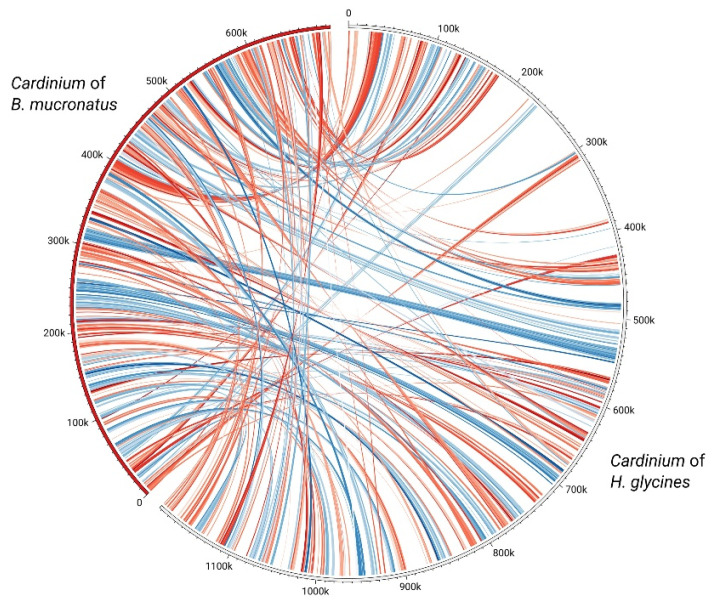
A plot showing the orthologs shared between the genomes of *Cardinium* of *Bursaphelenchus mucronatus* and *Cardinium* of *Heterodera glycines*. Coordinates are specified in bp.

In the *Cardinium* symbionts of *B. mucronatus* and *B. fraudulentus*, a pronounced enrichment in guanine over cytosine and thymine over adenine is observed across one half of the genome, with the opposite pattern in the other half (Figure 2A, inner track). This asymmetric nucleotide composition, known as GC skew, typically changes sign at genomic positions corresponding to the origin and terminus of DNA replication (Figure 2A, green arrows). In contrast, the *Cardinium* symbiont of *Heterodera glycines* also exhibits GC skew, although it is less pronounced (Figure 2B, innermost track), possibly reflecting subsequent genomic rearrangements that have altered replication-associated asymmetry. Furthermore, in both genomes, the putative origins of replication are located at positions distant from the *dnaA* gene, which is set at position zero on the circular genome map. It is also noteworthy that in both *Cardinium* genomes, the ribosomal RNA genes are not organized into a single operon but instead occur in two separate regions, corresponding to the 16S and 23S–5S rRNA loci (Figure 2, track 3, highlighted in red). As shown in Figure A5, the genomes of *Cardinium* associated with *B. mucronatus* and *H. glycines* exhibit extensive genomic rearrangements, indicating substantial structural divergence between these strains.

**Figure 3:**
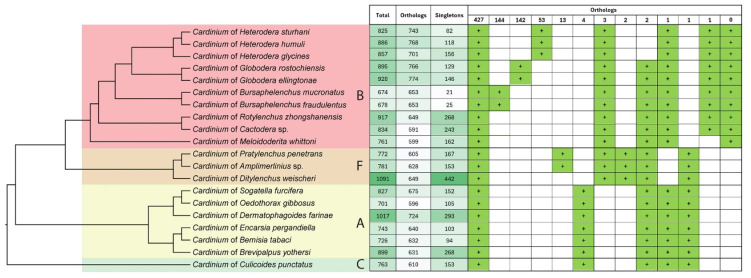
Distribution of total proteins, orthologue groups, and singletons encoded in twenty *Cardinium* genomes. Each column in the intersection matrix shows the presence of common orthologue clusters and their total number. The Bayesian 50% majority rule consensus tree as inferred from analysis of amino acid sequences of concatenation from eight protein-coding genes is given at the left. Capital letters and color areas indicate the *Cardinium* group.

The orthologous group (orthogroup) analysis of twenty *Cardinium* genomes is presented in Figure 3. These genomes share a total of 427 orthogroups (Table S2). The *Cardinium* genomes from nematodes contain between 21 (cBmuc) and 442 (cDwei) unique strain-specific genes (singletons), of which only 0–9% have an assigned function. All genomes from groups A, B, and C share only one orthogroup encoding a hypothetical protein. In addition, genomes from groups A, C, and F share a siderophore (surfactin) biosynthesis regulatory protein that is absent in the other groups. Genomes from groups B and F each possess three genes encoding unique proteins: a 57-amino acid hypothetical protein, ATP-dependent DNA helicase RecG, and N-acetylmuramoyl-L-alanine amidase. Although group B has no unique orthologues overall, the anhydromuropeptide permease (AmpG), a transporter protein located in the inner membrane of certain Gram-negative bacteria, becomes a unique orthologue for this group once the cMwhi genome is excluded.

Among *Cardinium* genomes from nematodes of different genera, shared orthologues vary substantially. Strains from *Heterodera* share 53 orthologues, including 51 hypothetical proteins, a low-specificity L-threonine aldolase, and a glycosyl transferase family 1 protein. Strains from *Globodera* share 142 orthologues, comprising 140 hypothetical proteins, phenylalanyl-tRNA synthetase β chain, and 1-acyl-sn-glycerol-3-phosphate acyltransferase. In contrast, *Bursaphelenchus*-associated *Cardinium* strains share only 144 hypothetical proteins (Figure 3).

The values of average nucleotide identity (ANI) and digital DNA-DNA hybridization (dDDH) based on a genome-to-genome sequence comparison calculated for *Cardinium* strains from plant-parasitic nematodes are shown in Table A3. Only three strain pairs, namely, cBmuc and cBfra, cGell and cGros, and cHhum and cHstu, are clearly above 95–96% (ANI) and 70% (DDH), used as boundaries for prokaryote species delineation, and, thus, these values indicate co-specificity of strains from these pairs.

**Figure 4:**
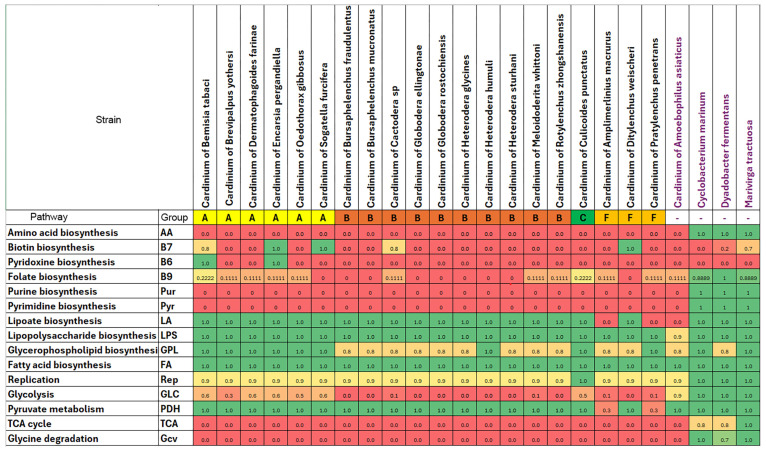
Distribution of reconstructed metabolic pathways and their completeness among genomes of *Cardinium* strains. Each number indicates a fraction of identified pathway genes (see Table S3 for more details).

Functional annotations of metabolic pathways in twenty *Cardinium* genomes, including ten new ones and four outgroup bacterial taxa, are given in Figure 4 and Table S3. Although the majority of *Cardinium* genomes lack biosynthetic pathways for all amino acids and nucleotides and most B vitamins, functional annotation indicates the presence of biosynthetic pathways for two B vitamins, pyridoxine (B6) and biotin (B7), in a small subset of genomes, while the lipoate coenzyme biosynthesis is conserved in all *Cardinium* genomes from groups A, B, and C and in one out of three group-F genomes. The unique distribution of the B7 biosynthetic pathway is described below, while the B6 biosynthesis genes were identified in two group-A *Cardinium* genomes. While the biosynthetic pathways for fatty acids and lipopolysaccharides are conserved in all analyzed genomes, the glycerophospholipid pathway is incomplete in eleven *Cardinium* genomes, with the absence of the *gpsA* gene encoding glycerol-3-phosphate dehydrogenase, a first pathway step converting glycerate-3-phosphate to glycerol-3-phosphate. We observed a significant reduction in the central carbohydrate metabolism pathways, namely, glycolysis/gluconeogenesis and the tricarboxylic acid (TCA) cycle, and conservation of the pyruvate dehydrogenase (PDH) enzymatic complex in all analyzed *Cardinium* except two group-F genomes. Finally, all *Cardinium* genomes lack catabolic or degradation pathways for carbohydrates and amino acids, confirming their metabolic dependence on carbon sources from the host.

**Figure 5:**
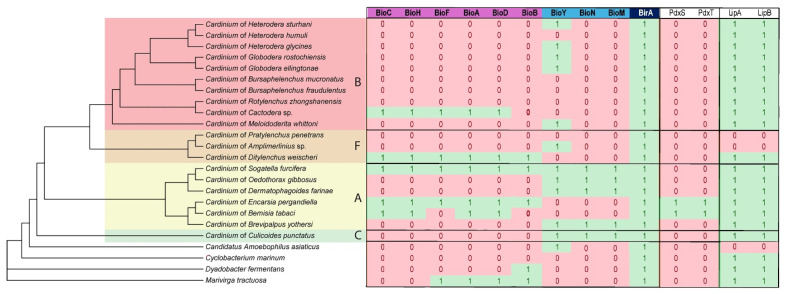
Presence (1) and absence (0) of genes of vitamin B7 (biotin), vitamin B6 (pyridoxal phosphate), and lipoic acid biosynthesis pathways in *Cardinium* and related bacteria. A Bayesian 50% majority rule consensus tree as inferred from the analysis of amino acid sequences of concatenation from eight protein-coding genes is given at the left. Capital letters and color areas indicate the *Cardinium* group.

**Figure 6:**
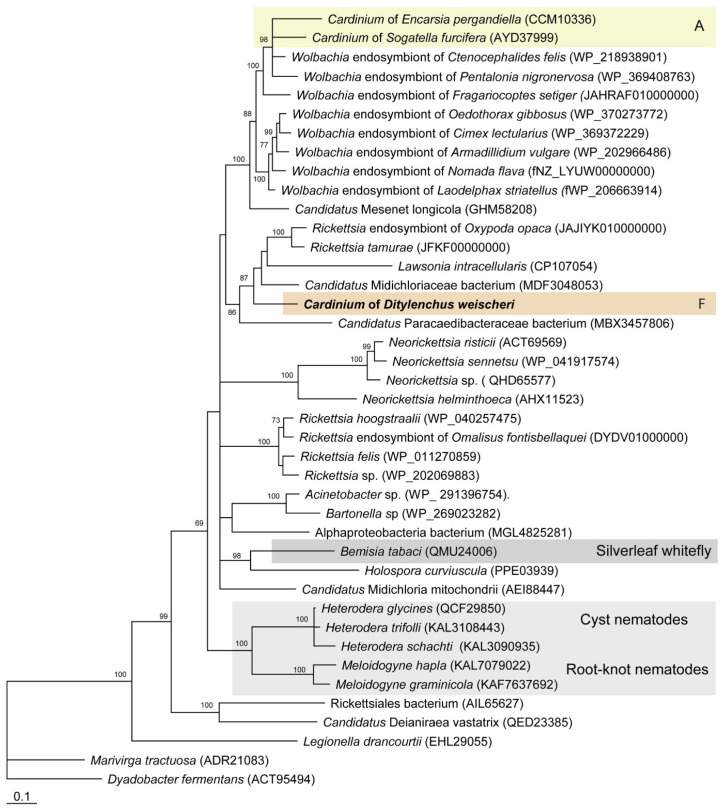
Phylogeny of the *bioB* gene from the biotin biosynthesis obtained from bacteria, nematodes, and whitefly, as inferred from the Bayesian analysis of amino acid sequence alignment (ntax = 41; nchar = 314). Posterior probabilities of more than 60% are shown at the branching points. The new sequence is given in bold. Capital letters and color areas indicate the *Cardinium* group.

**Figure A6:**
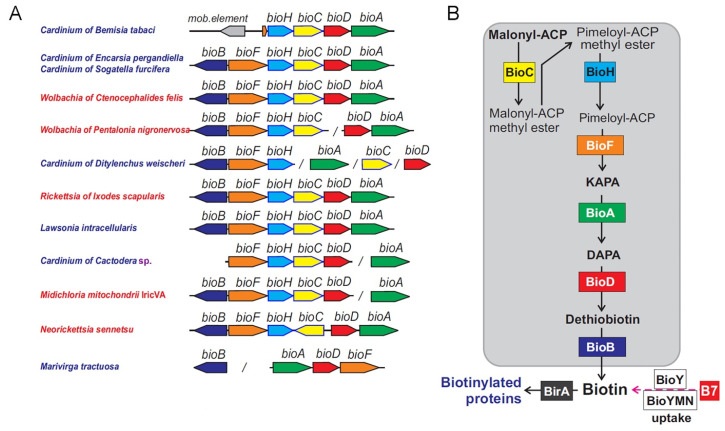
Genomic organization of the biotin biosynthesis pathway genes in *Cardinium* and some other bacteria (**A**) and schema of the biotin biosynthesis (**B**). *Cardinium* and related Bacteroidota species are shown in blue, while Rickettsiales species are in red.

**Figure A7:**
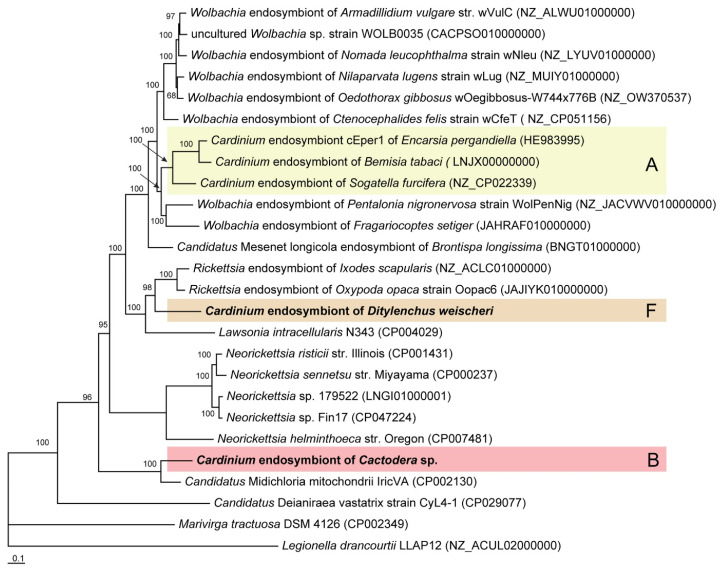
Phylogeny of the biotin synthesis pathway (concatenation of *bioA*, *bioB*, *bioD*, and *bioF* genes) as inferred from Bayesian analysis of amino acid sequence alignment (ntax = 26; nchar = 1373). Posterior probabilities of more than 70% are shown at branching points. *Legionella drancourtii* and *Marivirga tractuosa* are used as outgroups. New sequences are given in bold. Capital letters and color areas indicate the *Cardinium* group.

The biotin biosynthesis pathway is present in two genomes (cCsp3 and cDwei) and absent in other *Cardinium* strains from nematodes (Figure 4 and Figure 5). Within *Cardinium* from nematodes, a complete biotin pathway is found only in cDwei, and this pathway is only partially encoded in cCsp3. The *bioY* gene coding a membrane protein, which is part of the BioMNY ABC transporter system for biotin, is found in several *Cardinium* strains (cHstu, cHgTN10, cGros, cGell, cMwhi, and cAsp). In contrast, the biotin protein ligase BirA is present in all genomes, since it is an essential enzyme for the use of this cofactor by biotin-dependent enzymes from the fatty acid biosynthesis pathway (Figure 5). Genes involved in the biotin pathway are not arranged in one operon for cDwei (Figure A6). Phylogenetic analysis of the concatenation of *bioA*, *bioB*, *bioD*, and *bioF* genes for *Cardinium* and other bacteria indicated that these genes may undergo independent horizontal transfer events at least two times from distinct bacterial donors to *Cardinium* symbionts of plant-parasitic nematodes (Figure A7). The phylogeny of the *bioB* gene also confirms the independent origin of this gene for groups A and F and suggests an ancient origin of this gene for nematodes and its horizontal transfer from a rickettsia-like bacterium (Figure 6).

**Figure 7:**
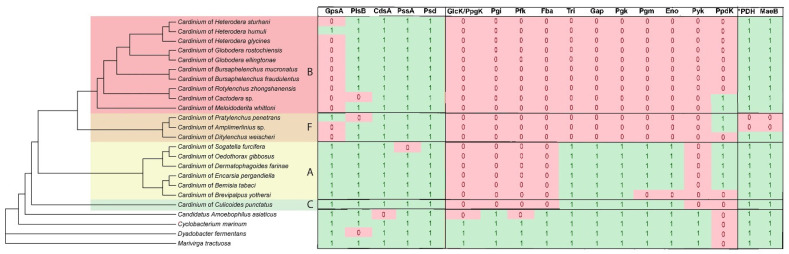
Presence (1) and absence (0) of genes of the glycerophospholipid biosynthesis pathway, glycolysis/gluconeogenesis, and pyruvate metabolism in *Cardinium* and related bacteria. A Bayesian 50% majority rule consensus tree as inferred from the analysis of amino acid sequences of concatenation from eight protein-coding genes is given at the left. Capital letters and color areas indicate the *Cardinium* group.

**Figure A8:**
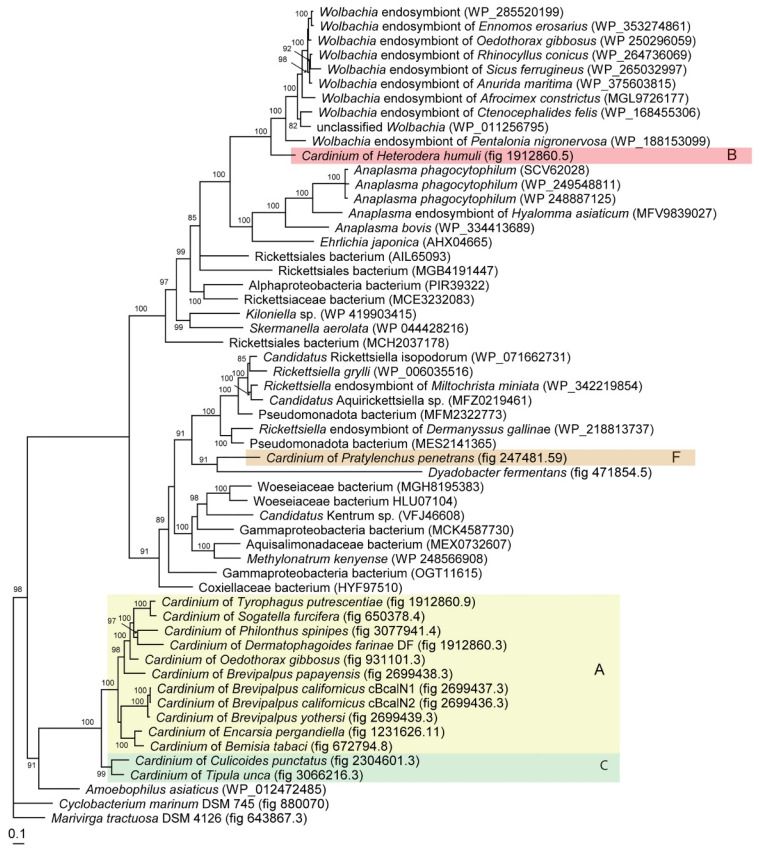
Phylogeny of the *gpsA* gene from the glycerophospholipid biosynthesis pathway as inferred from Bayesian analysis of amino acid sequence alignment (ntax = 57; nchar = 359). Posterior probabilities of more than 70% are shown at branching points. Capital letters and color areas indicate the *Cardinium* group.

**Figure A9:**
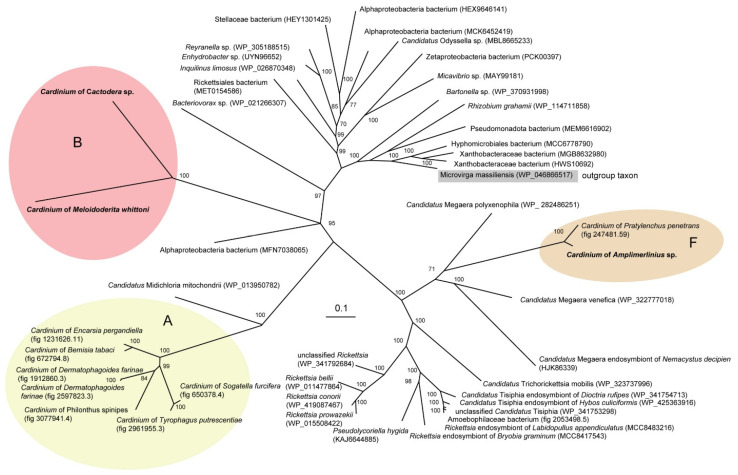
Phylogeny of the *ppdK* gene from gluconeogenesis as inferred from Bayesian analysis of amino acid sequence alignment (ntax = 46; nchar = 886). Posterior probabilities of more than 70% are shown at branching points. New sequences are given in bold. Capital letters and color areas indicate the *Cardinium* group.

The glycerophospholipid biosynthesis pathway is also incomplete in groups B and F, where the *gpsA* gene encoding the first pathway step is absent in most strains, except for cPpe and cHgTN10 (Figure 7). Phylogenetic analysis of *gpsA* amino acid sequences indicates that this gene was acquired independently in cPpe and cHgTN10 through horizontal gene transfer (HGT), rather than through close evolutionary relationships with the *gpsA* genes of groups A and C (Figure A8). The majority of core genes involved in glycolysis are also missing in *Cardinium* strains from nematodes. Phylogenetic analysis of the *ppdK* gene encoding the gluconeogenic enzyme pyruvate phosphate dikinase suggests that this gene was independently acquired by groups A, B, and F through horizontal transfer from at least three distinct bacterial donors (Figure A9). The *aceE*, *aceF*, and *lpdA* genes encoding the pyruvate dehydrogenase complex (PDH) form the only set of the central carbohydrate metabolism genes that is conserved in all *Cardinium* genomes from groups A, B, and C and is present in cDwei from group F. The malic MaeB enzyme converting malate to pyruvate is universally conserved in all *Cardinium* genomes.

Genes associated with DNA replication were found in all *Cardinium* genomes, except for *recN*, encoding the DNA repair protein RecN, which is present only in group C. The *polA* gene, which encodes DNA polymerase I and is a multifunctional enzyme essential for DNA replication and repair in prokaryotes, shows partial loss in several *Cardinium* strains. Specifically, deletions affecting fragments encoding the 5′→3′ polymerase domain (C-terminal region) and the 3′→5′ exonuclease domain (proofreading, central region) were detected in multiple strains from groups A and B and in one strain from group F (Figure A2). These gene reductions appear to have occurred independently during *Cardinium* evolution.

## 3. Discussion

Our findings highlight the remarkable diversity of *Cardinium* strains associated with plant-parasitic nematodes, expanding the known host range beyond previously reported species [7,12,13,17]. In particular, we report novel *Cardinium* associations in representatives of the nematode genera *Amplimerlinius*, *Bursaphelenchus*, *Ditylenchus*, and *Meloidoderita*, underscoring the broad phylogenetic and ecological distribution of this symbiont within plant-parasitic taxa. Plant-parasitic nematodes from these genera exhibit diverse feeding strategies and ecological behaviors, ranging from ecto- and endo-root parasites to wood inhabitants transmitted by insect vectors. A relatively larger number of *Cardinium* was found in the cyst nematodes (*Cactodera*, *Heterodera*, *Globodera*, and *Punctodera*), which are sedentary endoparasites that form long-lasting cysts in soil. Their infective juveniles invade plant roots and induce specialized feeding sites called syncytia. These genera are highly specialized and often target specific plants. Other *Cardinium* strains were presently reported from only one species of other nematode genera. *Meloidoderita* species are characterized by swollen, cystoid females with a functional stylet, vermiform males often lacking a stylet, and second-stage juveniles that induce syncytia in host roots. In the present classification, *Meloidoderita* and cyst nematodes belong to different orders or superfamilies. Stem nematodes of the genus *Ditylenchus* induce characteristic swellings, distortions, and hypertrophy in above-ground plant tissues, particularly stems, leaves, buds, and floral structures. These symptoms result from the nematodes’ feeding within the parenchyma, which disrupts normal cell development and triggers abnormal growth responses. *Ditylenchus weischeri*, in which *Cardinium* was found in the present study, is a highly specialized parasite associated with Canada thistle, *Cirsium arvense*. *Amplimerlinius* species are ectoparasitic nematodes that feed externally on root surfaces. While they are generally less destructive than endoparasites, they can impair root function, particularly under stress conditions or in high populations. Nematodes of the genus *Bursaphelenchus* represent a distinct ecological niche among plant-parasitic nematodes. Several species induce wilt diseases in various wood trees and palms. Some representatives of the genus combine plant parasitism with mycophagy (fungus-feeding) and rely on insect vectors. This dual feeding strategy and vector association make *Bursaphelenchus* ecologically unique in forest ecosystems [23].

Previous phylogenetic analyses based on limited *Cardinium* gene sequences identified the presence of two major lineages, or groups B and F, associated with plant-parasitic nematodes; however, the evolutionary relationship between these groups remained unresolved [7]. By incorporating a broader set of genes into the analysis, a clearer and more robust phylogenetic framework has emerged. This expanded dataset reveals that the two lineages are likely related and share a common ancestor, suggesting a single evolutionary origin followed by diversification within nematode-associated *Cardinium*. These findings refine our understanding of *Cardinium* evolution and suggest a potentially conserved symbiotic co-evolution within nematode hosts.

Symbionts belonging to the *Cardinium* clade are known to be vertically transmitted through maternal inheritance. However, the presence of closely related *Cardinium* strains in diverse and distantly related host species suggests that vertical transmission alone does not explain their distribution. This pattern has led to the conclusion that horizontal transmission events also play a role in the spread of these endosymbionts across different host lineages [11,24]. While horizontal transmission of *Cardinium* among arthropod hosts has been well documented in previous studies, this study represents the first comprehensive analysis of such patterns within plant-parasitic nematode groups. Cophylogenetic analyses of *Cardinium* strains and their plant-parasitic nematode hosts reveal that host and symbiont lineages have undergone correlated evolution, reflecting some degree of co-diversification. However, patterns of incongruence between host and symbiont phylogenies indicate evidence for several host switching events. These results expand our understanding of *Cardinium*’s evolutionary dynamics and suggest that cross-species transmission may play a significant role in shaping symbiont–host associations beyond arthropods.

The most widespread group within the *Cardinium* clade is group A, likely originating in soil mites, where it circulated extensively. From herbivorous mites, the symbiont spread to phloem-feeding insects with piercing–sucking mouthparts, enabling transmission to plants via salivary secretions. Experimental evidence confirms that *Cardinium* of insects can undergo interspecific horizontal transmission through plant tissue [25,26]. Subsequent evolutionary transitions led to its presence in endoparasitic wasps and predators such as spiders, rove beetles, and opilionids, likely via trophic interactions with infected hosts [7]. While there is no direct evidence that *Cardinium* is transmitted via plant tissue during nematode feeding, the hypothesis is biologically plausible and should be carefully experimentally tested. Discovering *Cardinium* in a wide range of plant-parasitic nematodes with diverse feeding strategies and ecological behaviors suggested that plants may serve as ecological reservoirs for *Cardinium* bacteria, facilitating their persistence in the environment and enabling horizontal transmission to new nematode hosts. This plant-mediated route may not only support the survival of *Cardinium* outside its primary host but also enhance its potential to colonize novel nematode hosts, contributing to the symbiont’s broad and scattered phylogenetic distribution.

Among the newly assembled *Cardinium* genomes obtained from plant nematodes in this study, two genomes originating from strains associated with wood-inhabiting *Bursaphelenchus* species exhibited remarkable genome reduction, with estimated sizes of approximately 695 kb. However, such a pronounced genome size reduction is not associated with extensive orthologous gene loss in these strains. This represents, to our knowledge, the smallest *Cardinium* genomes described to date. Interestingly, despite their extreme reduction, these genomes are comparable in size to those of certain genera and species of highly specialized endosymbiotic bacteria found in arthropods and nematodes [27,28].

Despite extensive genome reduction, *Cardinium* genomes retain a conserved core of DNA replication genes, including *dnaA*, *dnaB*, *dnaG*, and others, indicating a functional but streamlined replication apparatus. In contrast, genes involved in DNA repair, recombination, and replication checkpoint control are often incomplete or absent, reflecting increasing reliance on host cellular processes [29]. In several *Cardinium* strains, the *polA* gene encoding DNA polymerase I, a multifunctional enzyme that plays a key role in DNA replication and repair in prokaryotes, showed partial loss of domains. Loss of the proofreading exonuclease domain in family A DNA polymerase is well documented, whereas cases in which the polymerase domain itself is absent while exonuclease activity is retained are not well known [30,31].

Functional annotation of *Cardinium* genomes indicates the absence of or a reduction in several pathways, including the biotin biosynthetic pathway. Biotin, a vitamin belonging to the B-complex group, serves as a vital coenzyme that participates in fatty acid synthesis and other metabolic pathways in all bacteria. A complete biotin biosynthetic pathway, comprising *bioA*, *bioD*, *bioC*, *bioH*, *bioF*, and *bioB*, was identified in *Cardinium* cEper1 and *Cardinium* cSfur, endosymbionts that occur, respectively, in wasp *Encarsia pergandiella* [29] and planthopper *Sogatella furcifera* [24]. An incomplete biotin biosynthetic pathway, with losses of *bioB* and nearly all of *bioF*, was reported in the genome of *Cardinium* cBtQ1 from silverleaf whitefly, *Bemisia tabaci* [32]. In this study, a complete biotin synthesis pathway was found in the *Cardinium* strain from *D. weischeri*; only a partially encoded pathway was found in the *Cardinium* strain from *Cactodera* sp., and it was completely absent in all other newly sequenced *Cardinium*. The universal conservation of the biotin ligase gene *birA*, as well as of the fatty acid biosynthesis pathway, confirms that the biotin cofactor is universally indispensable in all *Cardinium* species and that most of them acquire this vitamin from the host.

The loss or absence of the biotin biosynthetic pathway in the majority of *Cardinium* strains suggests that endogenous synthesis of this vitamin is neither essential for the bacterium’s own metabolic requirements nor a critical factor in most *Cardinium*–host symbiotic relationships [8]. However, it is plausible that the biotin biosynthesis capabilities confer a nutritional advantage to their arthropod and nematode hosts. B vitamins, including biotin, are essential micronutrients that play critical roles in metabolic processes and overall host fitness. In such nutritional contexts, the ability of endosymbiotic bacteria to synthesize and supplement B vitamins may be crucial for host survival and reproductive success. Indeed, all *Cardinium* genomes lack biosynthetic pathways for the majority of B vitamins (with the only two exceptions of vitamins B6 and B7, as discussed above), suggesting the *Cardinium* species are multi-auxotrophs for nearly all indispensable B vitamins, thus, being totally dependent on their salvage from the host (and if it is a non-plant host, dependent on the host dietary supply).

Although biotin plays essential roles in metabolism, the capacity for de novo synthesis is limited to bacteria, plants, and some fungi, via a conserved four-step pathway beginning with pimeloyl-CoA and ending with biotin [33,34,35,36]. Animals, by contrast, lack this biosynthetic ability and rely on dietary intake [37,38]. Interestingly, biotin-related metabolism is dynamically regulated in cyst and root-knot nematodes and plant-parasitic nematodes, which have reacquired a bacterial-like biotin synthase gene through HGT [38]. While nematodes do not possess the full biosynthetic pathway, they retain the final step—conversion of dethiobiotin to biotin [39]—suggesting it may scavenge this metabolic precursor from the host to fulfill its biotin requirements and highlighting the nutrient’s functional importance in the nematode [38]. It has been well established that several genes involved in biotin biosynthesis, namely, *bioA*, *bioB*, and *bioD*, were horizontally transferred from bacteria into the genome of the whitefly (*Bemisia tabaci*) [40]. These horizontally acquired genes are believed to play an important role in supplementing the insect’s nutritional requirements, particularly given its nutrient-limited phloem-feeding lifestyle. Our analysis also confirms the presence of a horizontally transferred *bioB* gene in nematodes [38,41]. This finding suggests that nematodes, like whiteflies, may have independently acquired bacterial biotin-related genes, potentially enhancing their metabolic capabilities or ecological adaptability.

Phylogenetic analysis of the biotin gene cluster in endosymbiotic bacteria does not support previous hypotheses suggesting that these operons were acquired by *Wolbachia* through horizontal gene transfer from the genus *Cardinium* [42,43,44]. Instead, the data indicate a possible transfer from *Wolbachia* and related bacteria to *Cardinium*, as proposed by Zeng et al. [24]. The lack of phylogenetic congruence suggests that the acquisition of the biotin operon in *Cardinium* did not occur through a single ancestral event. Rather, it points to at least three independent horizontal transfer events throughout *Cardinium*’s evolutionary history: (i) endosymbionts of planthoppers, the whitefly *Encarsia* parasitoid wasp, and the silverleaf whitefly (group A); (ii) an endosymbiont of a stem nematode (group F); and (iii) an endosymbiont of a cyst nematode (group B). These instances of horizontal gene transfer likely originated from phylogenetically distinct bacterial donors.

It has been known that the highly conserved pathways of central metabolism are reduced or entirely absent in *Cardinium*. Moreover, our genomic searches did not find any catabolic pathway for carbohydrates or amino acids in *Cardinium* genomes. This indicates that *Cardinium* depends heavily on host-derived metabolites and pathway intermediates to compensate for its incomplete biosynthetic capabilities [8]. Analysis shows that most *Cardinium* genomes retain only a partial glycolytic pathway and, in the nematode-associated lineages, it is completely absent, suggesting an even higher degree of host dependence. Gluconeogenesis is similarly incomplete across *Cardinium*, and most strains lack the capacity to synthesize six-carbon sugars [8]. Glycerophospholipid metabolism appears to be incomplete in groups B and F, where the *gpsA* gene is missing. The GpsA enzyme normally catalyzes the reduction of glycerone-P (dihydroxyacetone phosphate, DHAP) to glycerol-P, a key precursor for glycerophospholipid biosynthesis. The absence of *gpsA* therefore disrupts the canonical entry point into the pathway. Interestingly, this pattern mirrors the presence of incomplete glycolysis observed in groups A and C. In these organisms, glycolysis does not proceed through the full set of enzymatic steps, yet glycerone-P remains one of the central intermediates that is still produced. The shared dependence on glycerone-P suggests a functional link between the truncated glycolytic pathway and the glycerophospholipid biosynthesis pathway.

There is strong phylogenetic and comparative genomic evidence that several glycolytic and carbohydrate metabolism enzyme genes have undergone horizontal gene transfer among bacteria. In *Cardinium* from the planthopper *S. furcifera*, for example, *gpml*, enolase, and *ppdK* were previously identified as HGT-derived genes acquired from distantly related proteobacterial donors [24]. These findings highlight the role of horizontal gene transfer in supplementing or restoring key metabolic functions in intracellular bacteria with reduced genomes. Our analysis further supports this pattern and reveals that *ppdK* was also horizontally acquired in *Cardinium* strains associated with nematodes. Notably, the phylogenetic placement of nematode-associated *ppdK* sequences indicates that these transfer events occurred independently from those reported in insect-associated *Cardinium.*

## 4. Materials and Methods

### 4.1. Nematode Populations, DNA Extraction, and Whole-Genome Amplification

Six nematode DNA samples containing *Cardinium* detected from our previous project [7] and several new nematode DNA samples were obtained and used in this study (Table A1). DNA from each sample was extracted from several adult and juvenile specimens using the proteinase K protocol. Nematodes were cut under a stereomicroscope. Cut nematodes in 16 μL water suspension were transferred into a 0.2 mL Eppendorf tube, and 3 μL proteinase K (600 μg mL^−1^) (Promega, Madison, WI, USA) with 2 μL 10 × PCR buffer (Taq PCR Core Kit, Qiagen, Germantown, MD, USA) was added to each tube. The tubes were incubated at 65 °C (1 h) and 95 °C (15 min) consecutively. Then, 2 or 3 μL from each sample in two or three replicates was submitted for whole-genome amplification (WGA). WGA was performed using an Illustra GenomiPhi V2 DNA amplification kit (Cytiva, Marlborough, MA, USA) following the manufacturer’s instructions. The replicates belonging to the same sample were mixed and purified using a QIAquick PCR Purification Kit (Qiagen, Germantown, MD, USA). A total of 30 μL containing 60 ng or more of DNA per μL was submitted for genome sequencing.

### 4.2. Genome Sequencing and Genome Assembly

DNA library construction and sequencing of samples was conducted by Novogene Co., Ltd. (Sacramento, CA, USA), and Dante Labs Inc. (New York, NY, USA). At Novogene, a NEBNext Ultra II DNA library prep kit from Illumina (New England Biolabs, Inc., Ipswich, MA, USA) was used following the manufacturer’s recommendations. Pooled DNA libraries were sequenced on an Illumina NovaSeq 6000 instrument to obtain 150 bp paired-end reads for three samples. At Dante Labs, an MGI library prep kit was used following the manufacturer’s recommendations. Pooled DNA libraries were sequenced on an MGI T7 instrument to obtain 150 bp paired-end reads for the samples. The quality of raw reads was evaluated using FastQC [45]. All genomes except *Bursaphelenchus fraudulentus* were assembled using SqueezeMeta v1.7.0 [46]. Assembly graphs were then manually checked with Bandage v0.8.1 [47] to remove short, low-coverage sequences not connected to the main graph. The *Cardinium* of the *B. fraudulentus* genome was assembled using the *Cardinium* of the *B. mucronatus* genome as a reference. In short, raw reads were cleaned using Trimmomatic v0.39 [48] and mapped to the reference genome using Bowtie2 v2.5.4 [49], followed by genome polishing using Polypolish v0.6.0 [50]. The same clean reads were then mapped to the genomic sequence obtained in the previous step using BWA v0.7.19 [51], followed by final polishing using Pilon v1.24 [52]. Genome completeness and contamination metrics were estimated by CheckM v1.2.4 [53]. Orthogroups between the genomes were found using OrthoFinder 3.0.1b1 [54]. The genome relatedness indices, viz., the average nucleotide identity (ANI) and digital DNA-DNA hybridization (dDDH) values, were calculated using JSpecies 1.2.1 [55] and GGDC 3.0 [56] tools, respectively. Genomes were visualized using DNAPlotter v18.2.0 [57]. A circular diagram of orthology between genomes was generated using the mummer2circos program [58].

The 16S rRNA, *atpD*, *dnaB*, *fusA*, *groEL*, *gyrB*, *infB*, *rspA*, and *sufB Cardinium* genes were assembled using corresponding reference genes of *Cardinium* of *H. humuli* (JAOPFT000000000.1) and *H. glycines* (CP029619) with Geneious 9.5 [59]. The D2–D3 expansion segments of the 28S rRNA gene for nematodes were also assembled with Geneious using published gene fragments of related species [60].

### 4.3. Phylogenetic Analysis of Bacterial and Nematode Genes

The new *Cardinium* gene sequences were aligned using ClustalX 1.83 [61] with their corresponding published gene sequences extracted from published genomes of selected *Cardinium* from GenBank [7,14,15,24,62,63]. Several alignments were generated: (i) 16S rRNA gene; (ii) *gyrB* gene; (iii) *atpD*, *dnaB*, *fusA*, *groEL*, *gyrB*, *infB*, *rspA*, and *sufB* genes; (iv) *bioA*, *bioB*, *bioD*, and *bioF* genes; (v) *bioB* gene; (vi) *polA* gene; (vii) *gpsA* gene; and (viii) *ppdK* gene. Outgroup taxa were chosen based on previously published data [7]. New and published D2–D3 expansion segments of 28S rRNA gene sequences of nematodes were also aligned using ClustalX. The best-fit models of DNA and protein evolution for sequence alignments were obtained using jModeltest [64] and ProtTest3.4.2 [65], respectively, with the Akaike Information Criterion. Nucleotide and amino acid sequence alignments were analyzed with Bayesian inference (BI) using MrBayes 3.1.2 and MrBayes 3.2.7, respectively [66]. BI analysis was initiated with a random starting tree and run with four chains for 1.0 × 10^6^ generations for nucleotide sequence alignment and for 1.0 × 10^4^ generation for amino acid sequence alignment. Two runs were performed for each analysis. The Markov chains were sampled at intervals of 100 generations. After discarding 10% and 25% burn-in samples for nucleotide and amino acid alignments, respectively, 50% majority rule consensus trees were generated. Posterior probabilities in percentage are given on the appropriate clades. The phylogenomic tree was inferred by JolyTree 2.1.211019ac [67]. For testing of alternative topologies, ML trees were reconstructed, and we used the Kishino–Hasegawa, Shimodaira–Hasegawa, and Shimodaira Approximately Unbiased tests as implemented in PAUP. 4.0 [68]. Trees were visualized with the TreeView 1.6.6 program [69] and drawn with Adobe Illustrator v.10. Cophylogenetic analysis was conducted to assess the evolutionary associations between host and symbiont lineages. Phylogenetic trees for the hosts and symbionts were constructed and imported in NEXUS format using the ape package in R. An association matrix was generated to indicate the presence or absence of each host–symbiont link. Pairwise phylogenetic distance matrices were calculated for hosts and symbionts using the cophenetic function. The Procrustean Approach to Cophylogeny (PACo) was implemented via the paco package, with a Cailliez correction applied to handle non-Euclidean distances. The significance of the global cophylogenetic signal was assessed using 1000 permutations, and residuals from the Procrustes analysis were intended to identify host–symbiont pairs contributing most to phylogenetic congruence [70,71].

### 4.4. Genome Annotation and Metabolic Reconstruction

New *Cardinium* genomes were initially automatically annotated by RAST [72], while the previously assembled genomes were downloaded from the Bacterial and Viral Bioinformatic Resource Center (BV-BRC) database [73], which also uses RAST for genome annotation. For functional gene annotation and reconstruction of metabolic pathways, we used a subsystems-based approach implemented in the SEED platform [74], which combines homology-based methods with three genome context techniques, namely, clustering of genes on the chromosome (operons), co-regulation of genes (regulons), and co-occurrence of genes in genomes. This approach allowed us to capture alternative biochemical routes (e.g., vitamin biosynthesis pathways implemented by different subsets of enzymes), as well as diverse nutrient transporters. For each metabolic pathway, we established genomic signatures represented by a subset of genes that are required for a complete pathway and determined the pathway completeness index by calculating the ratio of genes present in the genomes to the total number of pathway signature genes. The reference collection of bacterial metabolic pathways in the SEED database that was used for functional annotation of target genomes included biosynthetic pathways for all essential vitamins and cofactors, amino acids and nucleotides, and the central carbohydrate metabolism, as well as catabolic utilization pathways for ~100 carbohydrates, amino acids, and other carbon sources, such as lactate and short-chain fatty acids.

## 5. Conclusions

The newly assembled *Cardinium* genomes from diverse plant-parasitic nematodes broaden our understanding of the ecological and phylogenetic scope of this endosymbiont lineage. By clarifying the close evolutionary relationship of *Cardinium* groups B and F, this study supports a single origin for nematode-associated strains and highlights the extensive metabolic streamlining that characterizes these symbionts. The discovery of highly reduced *Cardinium* genomes in wood-associated *Bursaphelenchus* species, together with the patchy distribution of biotin biosynthesis and evidence for horizontal acquisition of key metabolic genes, underscores the dynamic evolutionary pressures shaping *Cardinium*–nematode associations. Future efforts integrating more complete genome assemblies with functional analyses of host–symbiont interactions will be critical for elucidating how metabolic dependency, genome reduction, and horizontal gene transfer jointly drive the evolution and ecological diversification of *Cardinium* across nematode hosts.

## Figures and Tables

**Table 1:** Assembly details and genome features for ten new strains of the *Cardinium* clade obtained in the present study.

**Strain** **Characteristics**	**cAmp**	**cBfra**	**cBmuc**	**cCsp3**	**cDwei**
NCBI BioSample number	SAMN51727604	SAMN51727605	SAMN51727606	SAMN51727607	SAMN51727608
Coverage (×)	91	644	190	1313	28
No. of scaffolds	73	1	1	12	49
Scaffold N50 (bp)	29,808	695,026	695,094	140,639	72,357
Assembly size (bp)	1,021,696	695,026	695,094	937,674	1,256,818
GC content (%)	37.99	41.07	41.07	38.71	35.23
No. of protein-coding genes	857	602	600	794	1025
No. of hypothetical proteins	436	261	259	409	576
No. of rRNAs	3	3	3	3	3
No. of tRNAs	38	35	35	36	38
No. of ncRNAs	10	3	3	3	2
CheckM completeness	68.29	69.29	69.29	71.48	72.02
CheckM contamination	4.69	0.55	0.55	1.37	3.21
**Strain** **Characteristics**	**cGell**	**cGros**	**cHstu**	**cMwhi**	**cRzho**
NCBI BioSample number	SAMN51727609	SAMN51727610	SAMN51727611	SAMN51727612	SAMN51727613
Coverage (×)	110	883	185	61	19
No. of scaffolds	84	105	19	53	144
Scaffold N50 (bp)	37,896	27,522	137,269	32,880	18,797
Assembly size (bp)	1,184,291	1,053,045	1,022,943	921,323	1,345,476
GC content (%)	37.64	37.68	38.69	36.84	36.53
No. of protein-coding genes	972	943	869	806	1061
No. of hypothetical proteins	565	564	460	408	621
No. of rRNAs	3	2	3	2	3
No. of tRNAs	37	37	36	36	36
No. of ncRNAs	2	2	4	3	3
CheckM completeness	70.38	69.84	69.84	71.29	69.02
CheckM contamination	2.09	1.00	0.55	0.82	2.46

cAmp—Cardinium of *Amplimerlinius* sp.; cBfra—Cardinium of *Bursaphylenchus fraudulentus*; cBmuc—Cardinium of *B. mucronatus*; cCsp3—Cardinium of *Cactodera* sp.; cDwei—Cardinium of *Ditylenchus weischeri*; cGell—Cardinium of *Globodera ellingtonae*; cGros—Cardinium of *G. rostochiensis*; cHstu—Cardinium of *H. sturhani*; cMwhi—Cardinium of *Meloidoderita whittoni*; cRzho—Cardinium of *Rotylenchus zhongshanensis*.

## Data Availability

Sequencing data are available from the NCBI database; accession numbers are indicated in Table 1, Table A1 and Table A2. All other relevant data are included within the manuscript.

## Supplementary Materials

The following supporting information can be downloaded at: https://www.mdpi.com/article/10.3390/ijms27021038/s1.

## Appendix A

**Table A1:** Plant-parasitic nematode species and *Cardinium* analyzed in this study.

Nematode Species	Nematode CDFA Sample Code or NCBI SRA Number	Location	*Cardinium* Strain Code	GenBank Accession Number	
				16S rRNA Gene of *Cardinium*	28S rRNA Gene of Nematodes
*Amplimerlinius* sp.	SRX25153634	China	cAmp	PX652261	PX647862
*Bursaphylenchus fraudulentus*	CD4140	Russia	cBfra	PX652264	PX647863
*B. mucronatus*	CD4127	Russia	cBmuc	PX652263	PX647864
*Cactodera rosae*	CD329	Mexico	-	PX652256	PX647860
*Cactodera* sp.	CD3612	Mexico	cCsp3	PX652260	PX647859
*Ditylenchus weischeri*	CD3221	Russia, Moscow region	cDwei	PX652262	PX647861
*Globodera ellingtonae*	CD3815b	Bolivia	cGell	PX652254	PX647858
*G. rostochiensis*	CD2568b	Bolivia, Cochabamba	cGros	PX652255	PX647857
*Heterodera latipons*	CD2047	Turkey, Kilis, Merkez	-	PX652258	PX647855
*H. sturhani*	CD2360a	China	cHstu	PX652257	PX647854
*Meloidoderita whittoni*	CD1799	USA, Florida	cMwhi	PX652265	KY704311
*Rotylenchus zhongshanensis*	SRX25153627	China	cRzho	PX652259	OM510443

**Table A2:** Protein-coding genes of *Cardinium* obtained and analyzed in this study.

*Cardinium* Strain	Nematode CDFA Sample Code or NCBI SRA Number	GenBank Accession Number							
		*atpD*	*dnaB*	*fusA*	*groEL*	*gyrB*	*infB*	*rspA*	*sufB*
*Cardinium* of *Amplimerlinius* sp. (cAmp)	SRX25153634	PX675661	PX654637	PX654650	PX675646	PX654656	PX654676	PX654689	PX654702
*Cardinium* of *Bursaphylenchus fraudulentus* (cBfra)	CD4140	PX675652	PX654628	PX654641	PX675637	PX654654	PX654667	PX654681	PX654693
*Cardinium* of *B. mucronatus* (cBmuc)	CD4127	PX675653	PX654629	PX654642	PX675638	PX654655	PX654668	PX654680	PX654694
*Cardinium* of *Cactodera rosae*	CD329	PX675656	PX654632	PX654645	PX675641	PX654663	PX654671	PX654684	PX654697
*Cardinium* of *Cactodera* sp. (cCsp3)	CD3612	PX675664	PX654640	PX654653	PX675649	PX654658	PX654679	PX654690	PX654705
*Cardinium* of *Ditylenchus weischeri* (cDwei)	CD3221	PX675662	PX654638	PX654651	PX675647	PX654657	PX654677	PX654692	PX654703
*Cardinium* of *Globodera ellingtonae* (cGell)	CD3815b	PX675655	PX654631	PX654644	PX675640	PX654661	PX654670	PX654683	PX654696
*Cardinium* of *G. rostochiensis* (cGros)	CD2568b	PX675654	PX654630	PX654643	PX675639	PX654662	PX654669	PX654682	PX654695
*Cardinium* of *Heterodera goettingiana*	CD3123a	PX675658	PX654634	PX654647	PX675643	PX654665	PX654673	PX654686	PX654699
*Cardinium* of *H. latipons*	CD2047	PX675659	PX654635	PX654648	PX675644	PX654666	PX654674	PX654687	PX654700
*Cardinium* of *H. sturhani* (cHstu)	CD2360a	PX675657	PX654633	PX654646	PX675642	PX654664	PX654672	PX654685	PX654698
*Cardinium* of *Meloidoderita whittoni* (cMwhi)	CD1799	PX675660	PX654636	PX654649	PX675645	PX654660	PX654675	PX654688	PX654701
*Cardinium* of *Rotylenchus zhongshanensis* (cRzho)	SRX25153627	PX675663	PX654639	PX654652	PX675648	PX654659	PX654678	PX654691	PX654704

**Table A3:** ANI (left) and dDDH (right) values for some *Cardinium* strains from plant-parasitic nematodes.

Strains	Nematode CDFA Sample Code or NCBI SRA Number	Strains												
		cAmp	cBfra	cBmuc	cCsp3	cDwei	cGell	cGros	cHgTN10	cHhum	cHstu	cMwhi	cPpe	cRzho
cAmp	SRX25153634		17.70	17.70	19.80	21.90	19.40	19.10	19.50	20.40	19.90	20.20	39.60	20.90
cBfra	CD4140	67.75		99.50	19.40	20.10	19.70	18.00	18.90	19.30	19.30	18.40	18.90	21.00
cBmuc	CDA4127	67.83	99.90		19.40	20.00	19.80	18.00	18.90	19.30	19.30	18.40	18.90	21.00
cCsp3	CD3612	69.63	66.94	66.99		20.80	20.50	21.10	20.60	21.50	21.00	22.10	20.70	18.80
cDwei	CD3221	77.6	67.51	67.57	69.07		21.20	19.90	21.60	20.30	21.00	19.00	22.30	19.20
cGell	CD3815b	71.66	67.68	67.75	70.7	70.91		79.30	22.30	22.80	22.40	19.00	20.00	20.90
cGros	CD2568b	71.63	67.72	67.76	70.69	71.08	97.55		22.60	22.80	22.40	18.10	19.60	20.10
cHgTN10	CP029619	70.42	67.04	67.09	68.51	69.59	77.47	77.47		55.00	54.20	18.50	18.30	19.90
cHhum	JAOPFT01	70.73	67.31	67.33	69.24	70.07	77.89	77.88	93.96		77.80	19.40	19.00	19.70
cHstu	CD2360a	70.76	67.21	67.25	68.72	69.91	77.85	77.78	93.97	97.52		20.10	19.10	19.80
cMwhi	CD1799	73.41	67.52	67.54	69.26	72.54	70.45	70.55	69.19	69.84	69.75		20.60	19.80
cPpe	RARA01	88.22	67.68	67.66	69.51	76.76	71.44	71.27	70.15	70.72	70.69	73.23		23.10
cRzho	SRX25153627	70.73	67.22	67.19	70.88	70.27	69.77	69.65	69.09	69.56	69.42	70.26	70.85	

cHgTN10—*Cardinium* of *Heterodera glycines*; cPpe—*Cardinium* of *Pratylenchus penetrans*. Values above the thresholds (95% for ANI, 70% for dDDH) for classification as the same species are marked in green.
